# Tolerogenic dendritic cells and negative vaccination in transplantation: from rodents to clinical trials

**DOI:** 10.3389/fimmu.2012.00218

**Published:** 2012-08-09

**Authors:** Aurélie Moreau, Emilie Varey, Gaëlle Bériou, Marcelo Hill, Laurence Bouchet-Delbos, Mercedes Segovia, Maria-Cristina Cuturi

**Affiliations:** ITUN INSERM 1064, CHU Hôtel-DieuNantes, France

**Keywords:** tolerogenic dendritic cells, transplantation, translational research, clinical trial, immune tolerance

## Abstract

The use of immunosuppressive (IS) drugs to treat transplant recipients has markedly reduced the incidence of acute rejection and early graft loss. However, such treatments have numerous adverse side effects and fail to prevent chronic allograft dysfunction. In this context, therapies based on the adoptive transfer of regulatory cells are promising strategies to induce indefinite transplant survival. The use of tolerogenic dendritic cells (DC) has shown great potential, as preliminary experiments in rodents have demonstrated that administration of tolerogenic DC prolongs graft survival. Recipient DC, Donor DC, or Donor Ag-pulsed recipient DC have been used in preclinical studies and administration of these cells with suboptimal immunosuppression increases their tolerogenic potential. We have demonstrated that autologous unpulsed tolerogenic DC injected in the presence of suboptimal immunosuppression are able to induce Ag-specific allograft tolerance. We derived similar tolerogenic DC in different animal models (mice and non-human primates) and confirmed their protective abilities *in vitro* and *in vivo*. The mechanisms involved in the tolerance induced by autologous tolerogenic DC were also investigated. With the aim of using autologous DC in kidney transplant patients, we have developed and characterized tolerogenic monocyte-derived DC in humans. In this review, we will discuss the preclinical studies and describe our recent results from the generation and characterization of tolerogenic monocyte-derived DC in humans for a clinical application. We will also discuss the limits and difficulties in translating preclinical experiments to theclinic.

## Introduction

The success rates of transplant surgery have significantly improved over the past fifty years. However, without treatment, the development of an immune response against the donor organ by the transplant patients leads to graft destruction. To block this immunological response and protect the transplanted organs from rejection, a range of general immunosuppressive drugs (IS) is necessary. Unfortunately, the use of IS drugs induces numerous adverse side effects, increasing the risks of infection and cancer (Dantal et al., 1998). The aim of research in transplantation today is to find an approach to induce long-term acceptance of transplants in the presence of minimal IS drug exposure. Cell therapy appears to be an innovative and promising strategy to address these problems (Bluestone et al., 2007). A European project called the “One Study” has been set up to test the efficacy of different immunoregulatory cell products in organ transplant recipients. In our center, tolerogenic dendritic cells (DC) will be injected into humans in an attempt to achieve donor-specific tolerance.

## Tolerogenic DC in animal models

DC are potent antigen-presenting cells (APC), able to induce either immunity or tolerance. After a brief description of the different types of mouse DC present *in vivo*, we will describe how tolerogenic DC can be derived *in vitro* in different animal models, and their efficacy in transplantation models. In the last part of this section, we will discuss the mechanisms of tolerance induced by TolDC.

### Different types of DC described *in vivo* in mice

DC are present in small numbers *in vivo* and are mainly localized in the spleen and lymph nodes (LNs). DC are a heterogeneous population of cells that can be classified into two main subsets: conventional DC and plasmacytoid DC. Conventional DC can be either resident or migratory cells.

Resident DC are present in the spleen, LNs and thymus. In the steady state, these DC are immature and become mature in the presence of danger signals. They can be divided into three subsets: CD4^+^CD8^−^, CD8α^+^ (DEC205^+^), and double negative, CD4^−^CD8^−^DC. They also differ in their methods of antigen (Ag) presentation. For example, CD8α^+^ resident DC are able to cross-present exogenous Ag on MHC Class I (den Haan et al., 2000). Thus, they mainly activate CD8^+^ T cells and produce high levels of IL-12, which leads to a type 1 response (Hochrein et al., 2001; Reis e Sousa et al., 1997). On the contrary, CD4^+^ resident DC present Ag on MHC Class II and mainly stimulate CD4^+^ T cells (Dudziak et al., 2007). In lymphoid organs, resident DC capture and present Ags to T cells. In contrast, migratory DC capture Ags in peripheral tissues and then migrate to LN where they present Ag to T cells. The most frequently described migratory DC are Langerhans cells present in the epidermis, although other migratory DC are also localized in the dermis and intestine. An inter-DC Ag transfer function was suggested by Allan et al. (Allan et al., 2006). In this context, migratory DC would bring Ag to LN, where resident CD8^+^ DC would efficiently present this Ag and induce CTL priming.

Plasmacytoid DC on the other hand, are actors of the immune response in the context of viral infections. These DC recognize viral DNA and RNA via TLR (Toll-Like Receptors) 7 and TLR9. Upon activation, plasmacytoid DC present Ag and produce high amounts of type 1 interferons.

In contrast to the different subsets of DC previously described, a last population of DC, called inflammatory DC (iDC), is not thought to exist in the steady state but to be produced *in vivo* in response to inflammation. A recent study by Cheong et al. showed that inflammatory DC originate in LN from circulating monocytes (Cheong et al., 2010b). Like the other DC, iDC are able to cross-present Ag by MHC Class I and stimulate naive or Ag-memory T cells (Cheong et al., 2010b). Interestingly, GM-CSF is essential for the generation of these DC as mice deficient in GM-CSF do not generate DC from monocytes in their spleen (Shortman and Naik, 2007).

### Generation of tolerogenic DC in animal models

The dogma described in the literature is that immature DC are tolerogenic and mature DC are immunogenic (Probst et al., 2003). However, some properties of mature cells, such as Ag presentation to T cells and *in vivo* migration to lymphoid organs, are also found in certain tolerogenic DC (TolDC). Thus, TolDC could be either immature, maturation resistant, or alternatively-activated cells (Ezzelarab and Thomson, 2011).

In most protocols, mouse DC are derived from bone marrow (BM). The conventional cytokines used to derive DC from precursors are GM-CSF and IL-4. However, a study performed in mice in 2000 showed that DC generated with low doses of GM-CSF in the absence of IL-4 have the properties of immature tolerogenic DC. These cells have a high capacity for Ag capture/presentation and induce a low level of allogeneic T cell proliferation. Furthermore, they are maturation-resistant and increase graft survival after *in vivo* injection (Lutz et al., 2000). Various DC manipulations *ex vivo* have been described to generate TolDC. For example, treatment of DC with Dexamethasone, VitaminD3, IL-10, TGF-β, rapamycin, LPS, or gene transfer (Morelli and Thomson, 2007) has been shown to increase their efficacy and block the maturation process (see Table 1 for details).

**Table 1:** **Examples of protocols used to generate BM-derived DC with tolerogenic properties in rodents—application in transplantation**.

	**Source**	**Name and Manipulations**	***In vitro* properties**	***In vivo* characteristics and effects**	**References**
**Cytokines/Growth factors**	Donor DC	**GM**^lo^**DC** [GM-CSF low dose]	– Maturation resistant (LPS, CD40L, TNF-α) – Allogeneic T-cell hyporesponsiveness	– Prolongation of heart allograft survival (>100 days) **superiority of **GM**^lo^**DC** in comparison to DC produced by both GM-CSF and IL-4 stimulation**	Lutz et al. (2000)
	Donor DC	**Alternatively activated DC - aaDC** [GM-CSF+Dex] followed by LPS activation	– 20-fold higher IL-10/IL-12 ratio in comparison to mature DC generated without Dex treatment – Allogeneic T-cell hyporesponsiveness, partial in response to secondary stimulation	– Increase of FoxP3^+^ expression in secondary lymphoid tissues – Prolongation of cardiac allograft survival by intravenous injection, but not by subcutaneous administration – Hyporesponsiveness of responder cells from animals injected with aaDC after *in vitro* restimulation	Emmer et al. (2006)
	Donor DC	**FLDC** [FIT3L]	– Immature phenotype and subdivided into pDC and cDC – mRNA expression of TGF-β, IL-10, and TNF-α	– Predominant homing to thymus (also in spleen or liver) – Induction of central tolerance (T cells clonal deletion) and peripheral tolerance (donor specific unresponsiveness) – Induction of donor-specific tolerance and long-term survival of skin allograft	Yamano et al. (2011)
	Recipient DC non pulsed	**Adherent DC-aBMDC** [GM-CSF+IL-4]	– Maturation resistant (LPS, CD40, poly l:C) – Allogeneic T-cell hyporesponsiveness	– Migration of syngenic aBMDC to the spleen **– Superiority of recipient-derived DC to prolong cardiac allograft survival in comparison to donor-derived DC (non plused)** – Diminution of humoral and cellular response and leukocyte infiltration in the heart after syngenic DC injection	Peche et al. (2005)
**Pharmacological mediators**	Donor DC (male)	**D_3_analog-conditionned DC** [GM-CSF+L-4+VitD_3_]	– Maturation resistant (anti-CD40, MCM, LPS)	– No sensitization of female hosts to male antigen – Prolongation of skin allograft survival	Griffin et al. (2001)
	Donor DC	**MRDC** [GM-CSF+L-4+VitD_3_] – purification of CD86^−^cells at the end of the culture	– Maturation resistant (DC1-maturation cocktail, LPS, agonistic CD40)	– Prolongation of cardiac allograft survival – Quick death of MRDC after injection – Reprocessing and presentation of donor-Ag from MRDC by host DC: **Donor-derived DC act as Ag-transporting cells**	Divito et al. (2010)
	Donor-Ag pulsed recipient DC	**RAPA-DC** [GM-CSF + IL-4+RAPA] – purification of CD11c^+^ cells at the end of the Culture – pulse with complex donor Ag	– Immature DC phenotype – Allogeneic T-cell hyporesponsiveness – Maturation resistant (anti-CD40) – Maintaining FoxP3^+^CD4^+^CD25^+^ Treg population *in vitro*	– Indefinite prolongation of cardiac allograft survival (>100 days) after donor-pulsed RAPA-DC injection and short rapamycin treatment – Graft infiltration by natural Treg cells	Turnquist et al. (2007)
**Genetic engineering**	Donor DC	**NF-κB ODN DC [GM-CSF + NF-κB ODN decoys]**	– Prevention of NO production in response to LPS-stimulation – Allogeneic T-cell hyporesponsiveness – Maturation resistant (LPS)	– Prolongation of cardiac allograft survival	Giannoukakis et al. (2000)
	Donor DC	**Ad CTLA4-lg-transduced ODN DC** [GM-CSF + Ad-CTLA-4lg + NF-κ B ODN decoys]	– Allogeneic T-cell hyporesponsiveness (superior to DCs treated only with NF-κ B ODN) – Induction of activated T-cell apoptosis	– Homing to T cells area of spleen – Indefinite cardiac allograft survival (40% of animals) – Donor-specific tolerance	Bonham et al. (2002)
	Donor DC	**FasL DC** [GM-CSF+IL-4 + hFasL cDNA in pBK-CMV vector]	– Low allogeneic T-cell proliferation due to apoptosis (FasL dependent manner)	– Allospecific hyporesponsiveness of splenocytes after FasL DC injection *in vivo* – Prolongation of cardiac allograft survival	Min et al. (2000)
	Donor DC	**vIL-10/CCR7-transduced DC** [IL-3 + IL-6 + SCF + murine CCR7 and viral IL-10 retroviral transduction] – Selection of transgene^+^ cells and culture with GM-CSF and irradiated syngenic BM cells	– Binding of CCL19-Fc by CCR7-transduced DC – Slighty lower expression of MHCII and CD80 in IL-10-transduced DC compared to CCR7-transduced DC – vIL-10/CCR7-transduced DC induce low allogeneic T-cell proliferation and IL-12 production	– Homing of CCR7-transduced DC to secondary lymphoid tissues (T cell zones) – Reduction of T cell proliferation and IFN-γ secretion *in vivo* after injection of vlL-10/CCR7-transduced DC (compared to CCR7-transduced DC) – Indefinite survival of cardiac transplantation after administration of vlL-10/CCR7-transduced DC – Donor-specific tolerance	Garrod et al. (2006)
	Donor DC	**RelB-silenced DC** [GM-CSF + IL-4 + RelB specific siRNA transfection]	– Maturation resistant (CD40L) – Failed to stimulate allogeneic T-cell responses	– Induction of Ag-specific immune suppression *in vivo* by KLH immunization – Indefinite cardiac allograft survival (50% of animals) – Ag-specific tolerence induction associated with increase of Foxp3^+^ Treg cells	Li et al. (2007)

Abbreviations: Ad Adenovirus BM Bone Marrow cDC conventional Dendritic Cell CTLA4-Ig Cytotoxic T-Lymphocyte Antigen 4-Antibodody fusion protein DC1 -maturation cocktail, contains IFN-γ, TNF-α, IL-1β, CpG, and poly I:C Dex dexamethasone Flt3L Fms-related tyrosine kinase 3 Ligand GM-CSF Granulocyte Macrophage-Colony Stimulating Factor KLH Keyhole Limpet Hemocyanin LN Lymph Node LPS Lipopolysaccharide MCM Macrophage-Conditioned Medium MRDC Maturation-Resistant Dendritic Cell NO Nitric Oxide ODN OligoDeoxyNucleotides pDC plasmacytoid Dendritic Cell poly I:C Polyinosinic:polycytidylic acid RAPA RAPAmycin SCF Stem Cell Factor TGF-β Transforming Growth Factor-β TNF -α: Tumor Necrosis Factor-α VitD_3_ Vitamin D3.

Modifications are provided as bold entries.

Compared to the different types of DC described *in vivo*, TolDC generated *in vitro* should be similar to inflammatory DC, as these cells are not normally found in the steady state but are present *in vivo* in a context of inflammation (Shortman and Naik, 2007). Furthermore, inflammatory DC need GM-CSF for their differentiation and this cytokine is also essential for the *in vitro* generation of TolDC.

While DC are derived from BM in rodents, monocytes are used in humans. To compare the importance of the precursors in the generation of tolerogenic DC, non-human primate models can be used. In most studies, DC are derived from peripheral blood monocytes. After CD14 positive selection, monocytes are cultured with GM-CSF (800–1000 U/ml) and IL-4 (500–1000 U/ml) to obtain DC (O'Doherty et al., 1997; Barratt-Boyes et al., 2000; Asiedu et al., 2002; Ashton-Chess and Blancho, 2005; Mortara et al., 2006; Zahorchak et al., 2007). In parallel, two studies have shown the possibility of deriving DC from CD34^+^ bone-marrow precursors (Pinchuk et al., 1999; Ashton-Chess and Blancho, 2005). Using cynomolgus macaques, we compared the generation of DC from monocytes and from BM (either from total cells as for rodents or from CD34^+^ precursors) (Moreau et al., 2008). Our results showed that the DC phenotype and function vary according to the origin of the precursors. As such, DC generated from monocytes (MoDC) have a more homogeneous phenotype with all cells expressing CD86. In BM derived DC, only half of the cells are CD86 positive, regardless of whether the CD34 precursors are isolated or not. However, neither MoDC nor BMDC express the maturation marker CD83, suggesting that these cells are semi-mature DC. In terms of their function, macaque MoDC induce less proliferation of freshly isolated natural Tregs than their BM-derived DC counterparts (Moreau et al., 2008). Another study performed in our center compared the generation of baboon DC from monocytes or from CD34^+^ BM precursors. The authors also concluded that different DC were obtained depending on the precursor cell-type (Ashton-Chess and Blancho, 2005).

### Efficacy of tolerogenic DC in animal models

In transplantation, DC present donor Ag to recipient T cells either by the direct pathway, the indirect pathway or the semi-direct pathway. By the direct allorecognition pathway, donor DC present donor peptide/donor MHC molecules to T cells, this type of Ag presentation is mainly associated with acute graft rejection. In contrast, the indirect pathway is defined by the presentation of donor peptide by recipient MHC molecules and is thought to induce chronic rejection. In the semi-direct allorecognition pathway, recipient DC present donor MHC molecules (transferred from donor cells) to T cells (Herrera et al., 2004; Smyth et al., 2006). In order to achieve donor-specific tolerance using DC therapy in transplantation, both donor tolerogenic DC (direct pathway) or recipient tolerogenic DC loaded with donor peptides (indirect pathway) have been tested in animal models of transplantation. The efficacy of these different types of DC has been demonstrated in rodent models, as described in Table 1 (Morelli and Thomson, 2007; Ezzelarab and Thomson, 2011).

Recently, Morelli's group demonstrated that injected *donor DC* are actually unable to directly regulate donor-specific T cells *in vivo* in mice. In fact, after injection, donor tolerogenic DC die quickly and the donor Ag is reprocessed and presented by the host DC via the indirect pathway (Divito et al., 2010). In this context, donor DC mediate their suppressive effects on T cells through endogenous conventional DC from the recipient mouse (Wang et al., 2012).

These results indicate that injected donor TolDC act as “donor Ag transporting cells”, which could be related to the DST (donor specific transfusion) protocol. DST, which consists in injecting donor blood into the recipient before transplantation, is still used in the clinic. Some studies have shown that DST improves graft survival and function (Sharma et al., 1997; Marti et al., 2006).

In parallel, we demonstrated in a rat model of fully MHC-mismatched cardiac allotransplantation that injection of *unpulsed recipient DC* the day before the transplant induces longer graft survival than the injection of donor DC (Peche et al., 2005). To improve the system and to create clinically applicable conditions, recipient DC were then injected into rats treated with a suboptimal dose of the IS drug, LF15-0195 (Beriou et al., 2005). This deoxyspergualin analog is known to inhibit DC maturation by blocking NF-κB activation (Yang et al., 2003). Both recipient DC and LF15-0915 have a synergic effect and this co-treatment induces tolerance to the allogeneic heart transplant in 90% of treated rats. We then investigated whether the tolerance was donor-specific. To answer this question, tolerant rats received syngeneic, donor or third-party skin grafts at 100 days post heart transplantation. Only the third-party skin graft was rejected, showing that the tolerance induced by recipient TolDC + LF 15-0195 was donor specific (Beriou et al., 2005).

To confirm the efficacy of cell therapy using recipient TolDC, we generated TolDC in mice (Segovia et al., 2011) and in non-human primates (Moreau et al., 2009). As previously shown in rats, injection of mouse recipient TolDC associated with a transient anti-CD3 treatment prolonged graft survival in both skin and pancreatic islet transplantation models (Segovia et al., in preparation). In macaques, we showed that TolDC are able to expand Treg *in vitro* (Moreau et al., 2008).

### Mechanisms of action of TolDC

TolDC are thought to exert their actions using different mechanisms. First, these cells can induce either T cell anergy or clonal deletion. T cell anergy occurs when DC lacking costimulation molecules interact with T cells. In the presence of Ag but without costimulatory signals, T cells become anergic and lose their ability to proliferate (Schwartz, 1997; Lechler et al., 2001). On the other hand, TolDC can induce T cell apoptosis. One mechanism described to induce this clonal deletion is an over-activation of T cells, called AICD (Activation Induced Cell Death). The Fas/Fas ligand pathway (Lu et al., 1997), but also expression of IDO (indoleamine 2,3-dioxygenase) (Mellor et al., 2003) by DC, leads to AICD in effector T cells. The T cells targeted by clonal deletion are either naive or memory cells (Kenna et al., 2008).

Another major mechanism of action of TolDC is the generation/expansion of regulatory T cells. Some studies have shown the ability of GM-CSF-derived DC to induce expansion of natural CD4^+^CD25^+^FoxP3^+^ Treg (Yamazaki et al., 2003; Emmer et al., 2006) whereas others have shown the ability of TolDC to generate Treg from naive CD4^+^CD25^−^ T cells (Fujita et al., 2007). In parallel, the generation of Tr1 by TolDC has also been demonstrated (Wakkach et al., 2003). Molecules expressed by TolDC, such as IDO or Galectin-1, have been shown or suggested respectively to be involved in the generation/expansion of regulatory T cells (Hill et al., 2007; Ilarregui et al., 2009). As the half-life of DC is short, the generation/expansion of Treg is an important mechanism. Indeed, Kendal et al. recently showed that Treg can maintain an infectious tolerance by *de novo* generation of Foxp3^+^ Tregs from naive CD4^+^ T cells (Kendal et al., 2011).

Besides the involvement of IDO expression by TolDC described in the two previous paragraphs, TolDC have also been shown to express tolerogenic markers such as HO-1 (Heme Oxygenase-1) and EBI3 (Epstein-Barr virus-Induced gene 3). Expression of HO-1 was demonstrated to correlate with DC maturation state (Chauveau et al., 2005) in that immature tolerogenic DC expressed high levels of HO-1, and this molecule enabled tolerogenic DC to inhibit allogeneic T cell proliferation. In both rats and macaques, blockade of HO-1 in TolDC impaired their ability to suppress T cell proliferation *in vitro*. Furthermore, in our model of tolerance to heart transplantation using both recipient TolDC and LF15-0195, blockade of HO-1 prevented tolerance induction (Moreau et al., 2009). EBI3^+^, another marker expressed by TolDC, also has a crucial role. In a rat cardiac allotransplantation model developed in the laboratory using syngeneic TolDC, an increase in double-negative T cells (TCRαβ^+^, CD3^+^, CD4^−^CD8^−^ NKRP1^−^, DNT) was observed in the spleen of tolerant mice. These DNT cells produced IFN-γ, which was essential for the tolerance induction, as anti-IFN-γ treatment of recipient mice led to the loss of tolerance induction (Hill et al., 2011). To investigate how injection of TolDC mediates IFN-γ production by DNT and tolerance induction, we identified the possible regulatory cytokines produced by TolDC. Our results showed that TolDC express EBI3. By using anti-EBI3 antibody and EBI3 siRNA, we demonstrated that expression of EBI3 by TolDC is essential for IFN-γ production by DNT cells. Furthermore, in our *in vivo* model of tolerance induction using TolDC, anti-EBI3 treatment of the recipient mice induced graft rejection, highlighting the key role of EBI3 expressed by TolDC in tolerance induction (Hill et al., 2011). It is important to note that the cytokine IL-35 is made up of EBI3 and p35 subunits. It has previously been demonstrated that IL-35 is secreted by regulatory T cells (iTr35^+^ cells) and contributes to their regulatory function (Collison et al., 2007; Niedbala et al., 2007; Collison et al., 2010; Chaturvedi et al., 2011).

As we had proved the relevance of using unpulsed recipient TolDC to induce donor-specific tolerance in several animal models, we wanted to understand the mechanisms of action of these cells. In contrast to most studies using TolDC (donor TolDC or donor-pulsed recipient TolDC), recipient TolDC were injected the day before transplantation (instead of one week before). After injection, recipient cells migrated rapidly to the spleen and were still detectable in this organ 15 days later (Peche et al., 2005). In parallel, donor derived MHC ClassII^+^ cells (OX3^+^) from the graft were present in the spleen 3–5 days post transplantation and seemed to interact with the injected TolDC. We hypothesized that injected recipient TolDC were able to process the donor Ag at this stage. To reinforce this hypothesis, we depleted graft passenger leukocytes (interstitial DC) from the donor hearts by administration of cyclophosphamide to the donor rat before transplantation. In this context, treatment of recipient animals with unpulsed recipient DC and LF15-0195 failed to induce any graft prolongation (unpublished results). However, the effect of recipient DC and LF15-0195 was rescued when donor splenic APC were injected in this model. These results highlight the essential role of graft passenger leukocytes in recipient TolDC therapy.

## Tolerogenic DC in humans and clinical trials

Studies performed in rodents ensured the characterization and the efficient use of TolDC *in vivo*. The goal today is to transfer this knowledge to humans in order to treat patients with tolerogenic DC. However, even though it is technically possible to derive DC from BM in humans (Berger et al., 2009), the culture of peripheral blood monocytes appears to be a reliable means to generate DC in humans. As described above, we know from studies in non-human primates that the different origin of tolerogenic DC in rodents and human can limit their comparison.

### Generation of human TolDC

Protocols of human MoDC generation are based on the knowledge acquired in animals. In most cases, human MoDC are obtained by culture of monocytes with GM-CSF and IL-4. However, more recently, human MoDC generated in the presence of GM-CSF and without IL-4 were described to have tolerogenic properties *in vitro*, like their counterparts in mice (Lutz et al., 2000; Chitta et al., 2008). Furthermore, as in animal models, other protocols have been reported to derive human tolerogenic DC from monocytes in the presence of pharmacological agents such as IL-10 or rapamycin (Morelli and Thomson, 2007; Gregori et al., 2010; Turnquist et al., 2010; Ezzelarab and Thomson, 2011).

To generate human TolDC for clinical trials, we decided to use a simple protocol. We derived human TolDC from monocytes (0.5 million/ml) cultured in AIM V medium supplemented with low-dose GM-CSF (100 U/ml) for 6 days. In this protocol, monocytes are enriched from leukapheresis of peripheral blood by elutriation (purity around 90–95%). Elutriation is a purification technique that separates cells based on their size and density (Berger et al., 2005). This cell separation technique enriches untouched monocytes in a closed and disposable system that is adapted for GMP (Good Manufacturing Practice) facilities. The advantages of using elutriation instead of bead selection are that the cells are untouched and there is no risk of injecting extra components (i.e., beads) to humans. The disadvantage of elutriation is a lower degree of cell purity, although this is not a real problem when autologous cells are injected. After one week of differentiation, human TolDC are more than 90% MHC-II^+^ and less than 2% contaminated with T cells, B cells, or NK cells. These TolDC are hypostimulatory and do not over-express CD80 or CD86 markers and remain CD83 negative after LPS/IFN-γ stimulation. Furthermore, upon stimulation, TolDC secrete very low doses of IL-12 but are able to produce IL-10. Interestingly, as we described previously in rats (Hill et al., 2011), human TolDC also express the tolerogenic marker EBI3 after stimulation. These results suggest that our protocol generates tolerogenic DC that are semi-resistant to maturation, which is essential to ensure that they will not mature and become immunogenic once injected into patients.

### Use of human TolDC in clinical trials

Even though clinical protocols of vaccination using immunogenic DC have been tested over the past 15 years to prevent the development of tumors in cancer patients (Correale et al., 2001; Redman et al., 2008), less is known about the potential use of tolerogenic DC in the clinic. A first study published in 2001 demonstrated the feasibility and safety of injecting autologous immature TolDC in healthy volunteers (Dhodapkar et al., 2001). In this study, immature DC were pulsed with peptides and injected by the subcutaneous route into two volunteers. Each individual received a single injection of 2 million cells. The DC injections were well-tolerated without signs of toxicity and no evidence of autoimmunity was detected. Injection of DC was associated with Ag-specific inhibition of effector T cell function and induction of Ag-specific CD8 Tregs *in vivo* (Dhodapkar et al., 2001; Dhodapkar and Steinman, 2002). The first phase I clinical trial using tolerogenic DC was reported recently in type 1 diabetic patients (Giannoukakis et al., 2011). Ten patients received four intradermal injections of 10 million autologous DC. Three patients received control DC generated in the presence of GM-CSF and IL-4 and seven patients received immunosuppressive DC generated in the presence of GM-CSF, IL-4, and antisense oligonucleotides targeting CD40, CD80, and CD86 transcripts. Use of tolerogenic DC generated with these antisense oligonucleotides was shown previously by the same team to have a preventive and curative effect on diabetes in NOD mice (Machen et al., 2004). This Phase I study demonstrated that intradermal injections of autologous TolDC (both control and immunosuppressive DC) are well-tolerated and safe in diabetic patients; no adverse effects or toxicity was observed. Interestingly, the authors observed a statistically significant increase in frequency of B220^+^CD11c^−^ lymphocytes in patients treated with autologous TolDC (both control and immunosuppressive DC) during the DC administration period compared to baseline (Giannoukakis et al., 2011). Other clinical trials in autoimmune diseases, and more specifically in rheumatoid arthritis (RA), will begin shortly. The first one will be performed by R. Thomas's team in Australia (University of Queensland). BAY11-7082-treated DC loaded with citrullinated peptides derived from candidate RA auto-antigens will be used (Hilkens et al., 2010). Indeed, in a mouse model of Ag-induced arthritis, the authors previously showed that injection of BAY11-7082 treated Ag-loaded DC suppressed DTH (Delayed Type Hypersensitivity) reactions and arthritis (Martin et al., 2007). BAY11-7082, aNFκB inhibitor, affects DC differentiation, leading to a low expression of MHC Class II and CD40. *In vivo* injection of BAY11-7082-treated DC prevents priming of immunity and induces IL-10 producing CD4^+^Tregs (Martin et al., 2003). In parallel, another clinical trial in RA will be performed by CMU Hilkens and JD Isaacs in the UK (University of Newcastle). In this case, autologous DC will be generated with Dexamethasone and VitaminD3 and loaded with synovial fluid (Hilkens et al., 2010).

So far there have been no reports of clinical trials using TolDC in transplantation. As part of a European project, we will test the safety of autologous monocyte-derived TolDC in kidney transplant patients.

### Advantages of using autologous TolDC

In animal models of transplantation, most studies use donor TolDC or recipient TolDC loaded with donor Ag. In contrast, we have shown the efficacy of unpulsed recipient TolDC to induce tolerance. In humans, the use of autologous TolDC is preferable due to the safety and feasibility of applying this type of DC to a clinic context.

In terms of safety, the major risk of donor TolDC injection in transplantation is donor sensitization. Maturation of TolDC after *in vivo* injection or the presence of a slight contaminant cell product could lead to the development of sensitization of the recipient to the donor Ag. In this case, priming or a higher immune response against the graft could potentially occur at the time of transplantation. Furthermore, another risk of injecting allogeneic cells is non-self recognition by the host immune system. In this context, the injected cells may be deleted by recipient NK cells (Yu et al., 2006).

In terms of clinical application in transplantation, the use of autologous TolDC is compatible with both living and deceased donor transplants. Autologous cell therapy could thus be applied to all transplanted organs. Another advantage of using autologous cell therapy is that the cell product could be prepared as soon as the patient is waiting for a transplant and preserved frozen. At the time of transplantation, the cells could be thawed and injected without any preliminary preparations. The use of autologous TolDC is all the more applicable to the clinic as neither the donor nor the time of transplantation have to be planned in advance, in accordance with the use of transplants from deceased donors.

Although the preparation and injection of autologous TolDC in patients would be costly, cell therapy is considered as a promising approach. It leads to an induction of Ag-specific tolerance without depleting an entire population of lymphocytes or blocking costimulation molecules. Like IS drugs, one could assume that these efficient but large-scale treatments could potentially induce side effects. Another cheaper alternative approach to induce Ag-specific tolerance would be to deliver donor Ags to quiescent conventional host DC *in vivo*. This technique was shown to be feasible in mice using either CD205^+^ DC or DCIR2^+^ DC (Hawiger et al., 2001; Bonifaz et al., 2002, 2004). In this second model, targeting of donor MHC molecules to DCIR2^+^ DC led to indefinite survival of MHC Class I mismatched skin grafts (Tanriver et al., 2010). However, it seems that the effect of Ag targeting to DC depends on their state of activation. For example, some studies have shown that injection of Ag coupled to DEC205 and anti-CD40 antibody or TLR ligands initiates immune responses against the targeted Ag (Bonifaz et al., 2004; Boscardin et al., 2006; Trumpfheller et al., 2008). So although this technique targets DC, the induction of tolerance or immunity will depend on whether the DC are immature or mature (Bonifaz et al., 2004). The use of human anti-human DEC205 Ab in vaccination was confirmed in human Ig-expressing transgenic mice (Cheong et al., 2010a). This technique would be useful on the strict condition that DC maturation can be inhibited, to assure that the Ags target only immature DC in humans (Shortman et al., 2009). In contrast, the first clinical trials with injected TolDC described above have proven the safety and absence of toxicity of using autologous DC in humans.

### Application of TolDC in the clinic

The cells that we described above were obtained from the blood of healthy volunteers. For the clinical trial in kidney transplant patients, TolDC will be generated using monocytes from patients with chronic renal failure. Before the beginning of the clinical trial, it is essential to validate our TolDC in these patients. A comparative study of the generation of clinical grade TolDC in healthy volunteers and in RA patients was reported prior to a clinical trial ongoing in RA using autologous TolDC (Harry et al., 2010). Their results showed that TolDC generated from RA patients have a similar phenotype and *in vitro* function as those generated from healthy controls (Harry et al., 2010). In order to develop immunotherapy for multiple sclerosis, another team described TolDC derived from relapsing-remitting multiple sclerosis (RR-MS) patients. Their results showed that TolDC generated with VitaminD3 from RR-MS patients and from healthy controls display a similar differentiation and function (Raiotach-Regue et al., 2012). As well as the origin of the samples (volunteers versus patients), other parameters have to be taken into consideration for the GMP preparation of TolDC, as described in Table 2.

**Table 2:** **DC preparation conditions**.

**Parameters of DC preparation**	**Controls to perform**
Optimal cell culture conditions	– patients sample – adequate cytokines and medium
Clinical grade reagents	– GMP grade cytokines and medium – closed systems as bags
GMP facility	– controlled room temperature/pressure – standardization and quality controls of the protocols – allowed and trained technicians
BASIC RULE = USE THE SIMPLEST PROTOCOL	

Prior to TolDC injection, different parameters which could influence immunogenicity and survival of the injected cells also have to be defined, as described in Table 3. One of these is the route of DC administration. Experiments performed in mice have shown that intravenous injection of Dex/LPS-treated BMDC prolongs cardiac transplant survival whereas subcutaneous injection of the same Dex/LPS-treated BMDC does not increase graft survival (Emmer et al., 2006). In parallel, our experiments in macaques show that intradermal injection of autologous TolDC prime an immune response while intravenous injection favors a tolerogenic role of these TolDC (unpublished results). A study also performed in monkeys confirmed the fact that intravenous injection of TolDC is well-tolerated (Zahorchak et al., 2007).

**Table 3:** **Parameters of DC injection**.

**Parameters of DC injections**	**Questions to answer before clinical trials**
Origin of DC	– donor DC – donor pulsed recipient DC – unpulsed recipient DC
Number of injections	– single – multiple
Time of DC injections	– Prior transplantation (day-7 or day-1) – Peri-transplantation – Post transplantation
Amount of cells administrated	related to the number of injections
Route of cell administration	– Intradermal or subcutaneous: inflammatory way – Intravenous: tolerogenic way
Associated treatments	e.g., Immunosuppressive drugs

Another parameter is the potential treatment associated with the cell injection, such as IS drugs. These drugs could either potentiate or inhibit the effect of TolDC *in vivo*. For the clinical trial in kidney transplantation, cell therapy will be performed in patients treated with several IS drugs. Previous studies have determined the interaction between DC therapy and IS. Indeed, our experiments in a model of transplantation have shown that treatment of rodents with rapamycin or cyclosporin A does not improve the TolDC effect. This is different from the injection of allo-Ag pulsed RAPA-DC in mice that promoted indefinite graft survival when treated with low doses of rapamycin at the time of transplantation (Turnquist et al., 2007). As regards human TolDC, some *in vitro* studies have shown that rapamycin increases CCR7 expression, which is necessary for TolDC migration to lymphoid organs (Sordi et al., 2006). Other IS, such as calcineurin inhibitors, including cyclosporin A or tacrolimus, block MHC-restricted Ag processing pathways in mouse BMDC *in vitro* (Lee et al., 2005). In the context of the One Study clinical trial, the patients will receive three IS in combination with the cell therapy: MMF (Mycophenolate mofetil), Tacrolimus and Prednisolone. From a safety point of view, it is necessary to validate that the TolDC will not interfere with the function of these IS. To answer this question, graft survival after injection of each IS with and without TolDC will be monitored in our mouse skin graft model. So far, we have observed that injection of MMF induces a prolongation of graft survival and injection of TolDC does not impair this effect. In fact, a slight increase in graft survival was detected (Segovia et al., in preparation). Similar experiments using the two other IS associated or not with DC therapy are ongoing. The combination of three IS in the presence or absence of cell therapy will be also tested.

## Conclusion

Cell therapy, e.g., TolDC, is currently considered as an attractive approach to minimize the use of IS in transplantation. Studies performed in rodent models have demonstrated the feasibility and efficacy of TolDC for the induction of tolerance in transplantation. In parallel, protocols to generate human TolDC *in vitro* have been defined but most have not yet been tested *in vivo*. New pre-clinical tools, such as humanized mice or non-human primates, have emerged and will be used to help translate the research findings from animal models to clinical application in humans.

### Conflict of interest statement

The authors declare that the research was conducted in the absence of any commercial or financial relationships that could be construed as a potential conflict of interest.
